# Enlargements of the Liver.—I

**Published:** 1910-01-01

**Authors:** 


### Medicine.

### ENLARGEMENTS OF THE LIVER.—I.

### THE PHYSICAL SIGNS OF HEPATIC ENLARGEMENT.

Neither a healthy liver nor one that is but slightly enlarged gives rise to any visible protuberance of the abdominal parietes. If the patient is thin, however, and the liver is considerably enlarged, it may be seen moving with respiration if the front of the abdomen is inspected in a good light. If the viscus is uniformly increased in size an abnormal prominence of the abdominal wall may be noticed in both the right hypochondriac and epigastric regions. If the hepatic enlargement involves the right lobe only, the abnormal fulness on inspection will be chiefly in the right hypochondrium, whilst if it is the left lobe that is most affected the fulness will be in the epigastrium. In detecting these abnormalities on inspection it is of considerable assistance if the patient makes steady respiratory movements of maximum excursion.

Local enlargements such as those due to tropical abscess, hydatid cyst, gumma, or malignant tumour, may cause a well marked local or nodular prominence in addition to a general tumefaction of the regions mentioned. In some cases of malignant disease, abscess or hydatid cyst, arising from the upper and outer part of the liver, local bulgings of the thorax have been noticed. Reddening and œdema of the skin over the corresponding region is sometimes present.

In some cases of considerable enlargement, when the abdominal wall is thin, the edge of the liver may be seen descending under the abdominal wall during inspiration and ascending during expiration. This may sometimes be a very important diagnostic sign, particularly well-marked when the enlargement is local or nodular.

More is to be learned about a liver by palpation than by any other single method of examination. It should be carefully borne in mind that the liver is not necessarily enlarged merely because it can be palpated. In a healthy person the left lobe may be felt in the upper part of the epigastrium and occasionally at the end of a deep inspiration the right lobe may be felt if the fingers are tucked in close under the right costal margin. In children, as a rule, the liver is normally of such a size as to be readily felt. In many patients, especially those suffering from emphysema and a wide epigastric angle, with or without bronchitis, the diaphragm descends abnormally low at the same time that the costal margin is abnormally raised, the result being that the normal liver becomes no longer covered by the ribs, and it is thus palpable though not enlarged.

When the liver is considerably enlarged it gives rise to a tumour occupying the right hypochondriac or the epigastric region, or both; when enormously enlarged, and especially if dislocated at the same time owing to dropping by reason of its weight, as in some cases of secondary malignant disease, its lower edge may extend below to the iliac crest and stretch across the abdomen below the level of the umbilicus into the left hypochondriac, or even the upper part of the left lumbar region.

The surface of the liver should be carefully examined with the hand, for important evidence as to the nature of the enlargement is sometimes to be obtained in that way.

The surface may be *smooth* in active and passive congestion, fatty degeneration, lardaceous disease, leucocythæmia, Hodgkin's disease, pernicious anæmia, unilobular cirrhosis, chronic biliary obstruction, suppurative pylephlebitis, suppurative cholangitis, and occasionally in diffuse carcinoma.

The surface feels *hard* in cases of carcinoma, cirrhosis, lardaceous disease, and passive congestion, nutmeg liver. In carcinoma it is almost of stony hardness.

The surface may be *uneven*, rough, or finely nodular in multilobular cirrhosis (hobnail liver) and primary malignant disease. Large nodules may be felt in cases of carcinoma, sarcoma, abscess, gumma, or hydatid cyst. In carcinoma the nodules vary considerably in size; they are hard, and in typical cases umbilicated. In sarcoma the nodules are less hard; if examined day by day they may be noticed to increase in size with rapidity. A gumma of the liver may be very large; it is not very hard, not umbilicated, and it is usually tender. A hydatid cyst, though containing fluid, is so tensely filled that it feels absolutely hard. It is very rare to get fluctuation in it, and almost as rare to obtain the hydatid thrill that has been described. The cyst feels like a solid tumour.

The surface is apt to be *tender* if the enlargement is due to congestion, abscess, cholangitis, gumma, carcinoma, sarcoma, perihepatitis, pylephlebitis, or cirrhosis.

Pulsation may be detected in some cases of nutmeg liver secondary to chronic valvular disease of the heart. A liver which is merely transmitting the pulsations either of the aorta or of the right side of the heart, often seems to be itself pulsating. A rare cause for a pulsatile liver occasionally met with is hepatic metastasis of a rapidly growing vascular sarcoma.

It is of great importance to note the position and character of the edge of the liver. This edge may be sharp and well-defined in cases of chronic congestion (nutmeg liver), lardaceous liver, unilobular cirrhosis, chronic biliary obstruction, and blood diseases such as leucocythæmia. It may be soft and indefinite in cases of active congestion and of fatty change. It may be finely nodular or beaded in cases of multilobular cirrhosis. It may be rounded and thickened in cases of perihepatitis or malignant disease. It may be curled up or curled under in cases of perihepatitis, the sign being diagnostic of the disease. With a distended gall bladder a rounded tumour may be felt projecting beneath the edge of the liver, though a Riedel's lobe may give very similar physical signs.

Percussion, whether simple or in association with auscultation, is one of the least trustworthy of the physical methods of liver examination. Palpation is far more valuable as a means of determining the lower limits of the organ. Unfortunately we have to rely upon percussion in determining the upper limits of the liver. The normal upper limits of absolute hepatic dulness correspond to the sixth rib in the right nipple line, the eighth rib in the right mid-axillary line, and the tenth rib in the line of the inferior angle of the right scapula behind. The lower limits of dulness are the costal margin in the right nipple line, the tenth rib in the right mid-axillary line, whilst behind it blends with the kidney dulness. If, however, the colon contains much gas the lower part of the hepatic region becomes resonant in front, even when the liver can be palpated below the costal margin.

If the liver is uniformly enlarged the area of absolute dulness is generally increased downwards, but it does not as a rule extend as low as the level of the edge of the liver, by reason of the varying degree of distension of the stomach and intestines with gas.

If there is a local enlargement of the liver arising from its upper surface, due to an abscess, for instance, or a growth, or a hydatid cyst, the dulness may encroach upon what should be pulmonary resonance; if carefully mapped out this increased dulness has sometimes been found to be dome-shaped.

To get an idea of the size of the liver the two points to lay most stress upon are: (1) the upper limit of the relative or deep dulness, and (2) the level at which the edge of the liver can be felt. In the right nipple line the vertical measurement between the two is normally 4½ inches.

*(To be continued.)*

